# Development of Silylated Lignin-Based Intumescent Flame Retardants for Biodegradable Plastics

**DOI:** 10.3390/polym17131727

**Published:** 2025-06-20

**Authors:** Heesu Yoo, Jaemin Jo, Sung Jin Kim, Bonwook Koo

**Affiliations:** 1 Major in Wood and Paper Science, School of Forestry, Science and Landscape Architecture, Kyungpook National University, 80, Daehak-ro, Buk-gu, Daegu 41566, Republic of Korea; aciertoryu@knu.ac.kr; 2 Institute of Agricultural Science and Technology, Kyungpook National University, 80, Daehak-ro, Buk-gu, Daegu 41566, Republic of Korea; jjm1234@knu.ac.kr; 3 Gumi Electronics & Information Technology Research Institute, Gumi 39171, Republic of Korea; sjkim@geri.re.kr

**Keywords:** flame retardant, lignin silylation, mechanical and fire properties, PLA composites

## Abstract

The global market for flame-retardant materials is expected to grow steadily, from USD 7.0 billion in 2022 to USD 16.6 billion in 2030, driven by increasing demand for environment-friendly fire safety solutions in transportation, construction, and electronics. Polylactic acid (PLA), a biodegradable polymer which possesses excellent mechanical properties, is increasingly being considered for future mobility applications. However, it is characterized by high heat release and toxic smoke during combustion, which are significant drawbacks. In order to address this, the chemical modification of Kraft lignin was achieved through a phenolation and subsequent silylation with tetraethoxysilane, aiming to mitigate the degradation of PLA’s mechanical properties while utilizing its inherent char-forming ability. The modified lignins were combined with ammonium polyphosphate (APP) and melt-mixed with PLA using an injection-mixing molder to prepare test specimens. Analysis by FT-IR, NMR spectroscopy, and SEM-EDS confirmed successful grafting of phenolic and silane functionalities, and thermogravimetric analysis demonstrated enhanced thermal stability of the modified lignins compared to unmodified ones. Vertical burning tests and limiting oxygen index (LOI) measurements showed that the PLA/APP/SPKL composite material achieved a V-0 UL-94 rating and 31.95% LOI, demonstrating the highest level of flame retardancy. This compares to the LOI of neat PLA, 19 to 21%. Despite the enhancement in flame retardancy to the V-0 level, the decline in tensile strength was limited, and the composite retained comparable mechanical strength to PLA-APP composites with V-2 flame retardancy. The findings indicate that the combination of phenolation and silylation of lignin with APP, a flame-retardant material, offers a viable and sustainable methodology for the fabrication of PLA composites that exhibit both flame retardancy and mechanical strength.

## 1. Introduction

Flame retardants (FRs) are defined as essential additives which are utilized to inhibit flame spread and reduce fire hazards in a variety of materials, particularly in automotive interiors, building insulation, and electronic housings. The global flame-retardant market is expanding rapidly, with an expected growth from USD 7.0 billion in 2022 to USD 16.6 billion by 2030, driven by increasing demand for sustainable and safe materials [1]. However, conventional halogenated FRs, such as brominated and chlorinated compounds, release toxic gases, including dioxins and furans, during the combustion. This has significant implications for both the environment and human health, as it poses a range of severe risks. Consequently, non-halogenated alternatives have attracted considerable attention in recent years, particularly regarding fire safety concerns in electric vehicles and battery systems [2,3,4].

Polylactic acid (PLA) is a biodegradable polymer which has a wide range of applications in various fields such as packaging materials, fibers, electronic products, and automotive parts due to its excellent mechanical strength and biodegradability. However, PLA exhibits inherent limitations, including low flame resistance, high flammability, and melt flow under heat, and its limiting oxygen index (LOI) is known as around 20% [5,6]. These drawbacks significantly restrict the application of PLA in environments with high fire risks. To address this issue, recent studies have explored the incorporation of various bio-based additives, such as cellulose, pectin, graphene, chitosan, and lignin, to enhance the flame retardancy of PLA [7,8,9,10].

Lignin, a natural aromatic polymer, is a by-product of the pulp process, and it is produced in such quantities as to exceed 100 million tons per annum on a global scale. The lignin under discussion is distinguished by its carbon-neutral and biodegradable properties, and it contains numerous aromatic rings and hydroxyl groups for chemical modification. Its high carbon content, reaching up to 60%, contributes to the formation of a char layer during combustion, thereby enabling it to act as a carbon source in intumescent flame retardant systems [11]. However, lignin exhibits significant heterogeneity, thus necessitating chemical modification for its compounding with other materials [12]. Phenolation has been shown to enhance its reactivity by enriching hydroxyl groups and facilitating the introduction of new reactants [13]. Furthermore, changes in the mechanical properties of lignin, such as an improved surface activity and polarity due to reduced polydispersity, enhance its applicability and compatibility as a composite material [14]. The silylation process, conducted for the purpose of enhancing the compatibility of lignin, adjusts the polarity of the lignin added to the composite material, thereby increasing compatibility. Concurrently, a silica network is established in order to enhance thermal stability and mechanical properties [15].

Flame retardants that use lignin alone do not achieve excellent flame resistance, and therefore they are often mixed with various flame-retardant additives [11,16]. Phosphate-based additives are extensively applied in the development of lignin-based flame retardants due to their numerous advantages, including being halogen-free and having low toxicity and high efficiency. The most prevalent phosphate-based additive applied for the purpose of flame retardancy in biomass is ammonium polyphosphate, which, in the course of combustion, functions as an acid and gas source, leading to charring and, by extension, smoke reduction and enhanced thermal stability [17].

In contrast to the high concentrations of kraft lignin (KL) and APP utilized in previous studies [16,18,19,20], this study employed a dual modification strategy involving phenolation and silylation of lignin, thereby achieving a simultaneous balance between flame retardancy and mechanical properties. Phenolation using ammonium polyphosphate (APP), the most widely used phosphate-based flame retardant in halogen-free and intumescent flame-retardant systems, increases the free phenol groups within the lignin molecules, thereby enhancing the interface interaction with the PLA matrix. This will increase the thermal decomposition start temperature (T_5_) of the composite and improve dispersibility, thereby suppressing the formation of fine agglomerates in the blend. The process of silylation, employing ethyl silicate, results in the deposition of silica nanoparticles onto the surface of lignin. This process effectively impedes direct molecular chain interactions between lignin molecules, thereby mitigating interface concentration clusters and defects. Finally, this study aims to develop flame-retardant PLA composites with improved thermal and mechanical properties by uniquely combining phenolic and silylated lignin with APP.

## 2. Materials and Methods

### 2.1. Materials

Kraft lignin (KL), serving as the carbon source for lignin-based hybrid flame retardants, was obtained from Sigma-Aldrich (St. Louis, MO, USA). The phosphorus-based additive, ammonium polyphosphate (APP, degree of polymerization ≥ 1000), was supplied by Wellchem Industry (Quzhou, Zhejiang, China), and the silicon-based additive, ethyl silicate (98%), was purchased from Daejung Chemicals & Metals (Seoul, Republic of Korea). Polylactic acid (PLA), used as the polymeric matrix, was kindly provided by Ecomass (Incheon, Republic of Korea).

For the phenolation of KL, phenol (>99.5%, TCI, Tokyo, Japan), sulfuric acid (98%, Sigma-Aldrich, USA), and ethyl acetate (99%, Daejung Chemicals & Metals, Seoul, Republic of Korea) were used; phenolated kraft lignin was designated PKL. Silylation of PKL to obtain the silylated PKL (SPKL) was carried out by first preparing a precursor solution of ethanol (99.5%, Duksan, Seoul, Republic of Korea) and acetic acid (99.5%, Duksan, Seoul, Republic of Korea), into which ethyl silicate was introduced under controlled conditions.

### 2.2. Phenolation of Kraft Lignin (PKL)

The phenolation of lignin was carried out following the protocol of Jiang et al. (2018) and Ou et al. (2022) [13,21]. In brief, 5 g of Kraft lignin and 15 g of phenol, with a weight ratio of lignin to phenol of 1 to 3, were charged into a three-neck round-bottom flask. Prior to addition, the phenol was melted in a thermostatted water bath at 70 °C, then was charged, and sulfuric acid (5 wt% relative to lignin) was introduced as a catalyst. The reaction was maintained at 120 °C for 2 h under magnetic stirring at 250 rpm. Upon completion, the mixture was rapidly cooled with running tap water, and the phenolated Kraft lignin (PKL) was recovered by dissolution in 500 mL of ethyl acetate. Residual reagents were removed by washing the PKL with distilled water until the filtrate reached pH 7, and the purified PKL was then stored in a vacuum oven at 70 °C until further analysis and silylation.

### 2.3. Silylation of Phenolated Kraft Lignin (SPKL)

**Figure 1:**
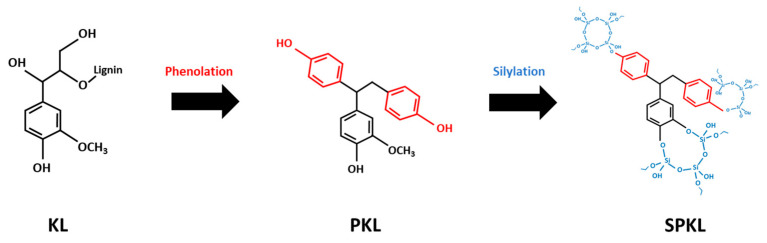
Reaction scheme of phenolation and silylation of KL.

The silylation of PKL was carried out according to the procedure reported by Liu et al. (2022) [22] (Figure 1). To prevent moisture uptake, PKL stored in a vacuum oven was kept in a desiccator until use. A precursor solution was prepared by combining ethyl silicate, ethanol, distilled water, and acetic acid in a molar ratio of 1:4:4:0.01, and stirring the mixture for 5 min. The PKL was then immersed in this precursor solution and reacted at 35 °C for 4 h under magnetic stirring at 400 rpm. Upon completion, the final modified lignin was recovered by vacuum filtration and washed repeatedly with distilled water. The sol–gel-derived silylated PKL (SPKL) was finally dried in a vacuum oven at 70 °C for 24 h.

### 2.4. Preparation of PLA, APP, and Modified KL Composites with Intumescent Flame Retardancy

**Figure 2:**
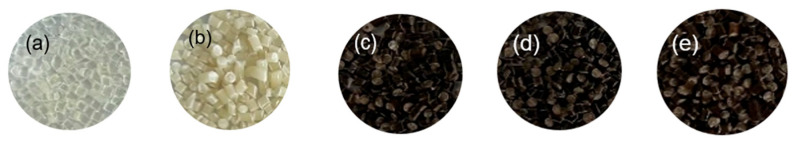
PLA composites with different compositions ((**a**): PLA, (**b**): PLA/APP, (**c**): PLA/APP/KL, (**d**): PLA/APP/PKL, (**e**): PLA/APP/SPKL).

Prior to composite preparation, PLA, APP, KL, PKL, and SPKL were dried in a vacuum oven at 60 °C for at least 8 h to remove residual moisture. The composite was prepared by hot-melt extrusion at 180 °C using a Minimax Molder (Custom Scientific Instruments, Easton, PA, USA). Once the PLA was fully molten, APP, KL, PKL, or SPKL were added at the ratios given in Table 1 and extruded under shear (CSI set to 60% of maximum output voltage, applied pressure 400 kPa) for 8 min until a visually homogeneous dispersion was achieved, after which the injection-molded plastic composite was cut into the form shown in Figure 2.

The extrudate was then pelletized by compression molding on a hot press (QM900S, Qmesys Co., Ltd., Gwangmyeong, Republic of Korea) using a 128 × 13 mm mold with a 3 mm gap. Molding was performed at 180 °C under 8 MPa for 5 min. The compositions of the flame-retardant PLA composites are listed in Table 1, and photographs of the prepared pellets are shown in Figure 2.

### 2.5. Characterization of Modified Lignin and Composites

Fourier-transform infrared spectroscopy (FT-IR; ALPHA, Bruker, Karlsruhe, Germany) was used to determine the functional groups introduced by phenolation and silylation of lignin. Spectra were recorded over the 4000–400 cm^−1^ range with a spectral resolution of 4 cm^−1^ and 32 scans per sample. Furthermore, the functional groups resulting from the silylation of lignin were determined by means of a solid-state ^29^Si HPDEC-MAS NMR spectroscopy. The NMR spectra were recorded on a Bruker AvanceNeo 500 MHz spectrometer equipped with a 4 mm HPDEC MAS probe, spinning at 14 kHz. The ^29^Si resonance frequency was 99.38 MHz. Cross-polarization experiments employed a contact time of 2 ms, while direct-excitation (HPDEC) measurements used a 90° pulse length of 6.5 µs and a relaxation delay of 30 s between scans.

Morphological characterization of the modification was conducted using field-emission scanning electron microscopy (FE-SEM; JEOL, Tokyo, Japan). The operating conditions were set to an accelerating voltage of 5 kV, an emission current of 10 µA, and a working distance of approximately 8 mm. Prior to imaging, samples were sputter-coated with platinum to avoid surface charging. In addition, elemental composition analysis was performed using an energy-dispersive X-ray spectroscopy (EDS) detector attached to the FE-SEM.

UL-94 vertical burning tests were carried out in accordance with ASTM D3801 to evaluate the flame retardancy of the lignin-added PLA composites [23]. Specimens (128 × 13 × 3 mm) were vertically mounted so that their lower edge sat 20 mm below a standard butane flame. Flame-spread rating, molten-drip behavior during the final 10 s of flame exposure, and ignition of absorbent cotton placed beneath each specimen were recorded. Limiting oxygen index (LOI) measurements followed ASTM D2863, using specimens of 70 × 6.5 × 3 mm in a specialized LOI chamber to determine the minimum oxygen concentration required to sustain combustion [24].

Thermal stability of the composites was assessed by thermogravimetric analysis (AUTO-TGA, TA Instruments, New Castle, DE, USA) under nitrogen and oxygen environments, each at a flow rate of 40 mL/min. Samples were heated from 100 °C to 800 °C at a rate of 10 °C/min, yielding TGA and DTG profiles. From these, the temperatures corresponding to 5% and 50% weight loss (T_5_ and T_50_) and the maximum decomposition temperature (T_max_) were determined.

Tensile strength testing adhered to ASTM D638, employing a universal testing machine (Hounsfield H500M, Redhill, UK) at a crosshead speed of 10 mm/min [25]. Each formulation was tested on at least three specimens, and the mean tensile strength and elongation value were reported.

## 3. Results and Discussion

### 3.1. Chemical Characterization of Modified KL

**Figure 3:**
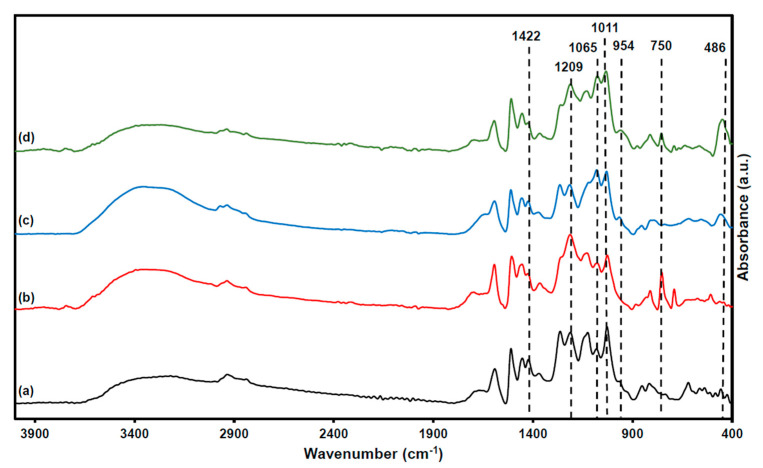
FT-IR spectra of KL (**a**), PKL (**b**), SKL (**c**), and SPKL (**d**).

To determine the chemical changes imparted by phenolation and subsequent silylation of Kraft lignin (KL), the FT-IR spectra of unmodified and modified lignins were compared (Figure 3).

Phenolation of KL resulted in a number of characteristic spectroscopic alterations [26,27]. The peaks at 1422 cm^−1^ and 1011 cm^−1^ assigned, respectively, to the asymmetric C–H deformation of methylene bridges and the in-plane deformation of aromatic C–H bonds, exhibited clear intensity alterations, consistent with successful phenolic substitution. Notably, an increased absorbance at 1209 cm^−1^ was observed, attributable to the stretching vibration of phenolic C–O bonds introduced by the grafted phenol groups. A new band at 750 cm^−1^ also appeared; this peak arises from out-of-plane C–H bending vibrations in substituted phenolic rings, reflecting the reaction between the phenol’s ortho/para positions and lignin’s α-hydroxyl moieties, and thus confirms effective phenol incorporation into the lignin structure.

Silylation of PKL was likewise confirmed by the emergence of characteristic Si–O vibrational bands [28]. The strong, broad absorption band at ~1060–1065 cm^−1^ characteristic of nano-SiO_2_ corresponds to the asymmetric and symmetric stretching vibrations and shear modes of Si–O–Si linkages, while the peak at 954 cm^−1^ is due to asymmetric Si–OH stretching, and the feature at 486 cm^−1^ arises from Si–O bending modes [22,29,30,31]. These signals demonstrate successful formation of a silica network on the lignin backbone via the sol–gel silylation process [32].

**Figure 4:**
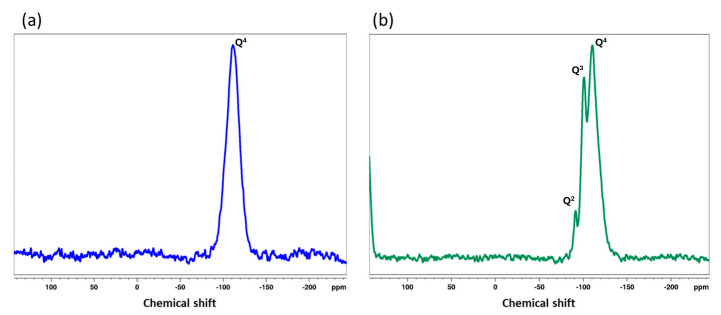
Solid state ^29^Si HPDEC-MAS NMR spectrum for the (**a**) SKL and (**b**) SPKL.

Solid-state ^29^Si HPDEC-MAS NMR spectroscopy was used to further elucidate the structures of PKL and SPKL (Figure 4). Three distinct resonances corresponding to the Q^2^ [Si(OSi)_2_(OH)_2_], Q^3^ [Si(OSi)_3_(OH)], and Q^4^ [Si(OSi)_4_] structural units within the silica network were observed. The Q^2^ site, characterized by two siloxane bonds and two hydroxyl groups, typically represents the outermost surface of the silica nanoparticles. The Q^3^ site, which has three siloxane bonds and one Si–OH group, is also located on or near the particle surface. The Q^4^ site, where the silicon atom is connected to four neighboring silicon atoms through oxygen bridges, reflects a fully condensed, highly cross-linked silica network [33].

In the SKL spectrum, only a broad Q^4^ resonance centered at −111.55 ppm was detected, indicating the formation of a pure physical silica coating. In contrast, the SPKL spectrum exhibited three well-resolved peaks at −91.29 ppm (Q^2^), −101.23 ppm (Q^3^), and −110.50 ppm (Q^4^). The appearance of Q^2^ and Q^3^ signals in SPKL confirms the formation of chemical bonds between silanol groups and the phenolic hydroxyl groups on the lignin backbone.

**Figure 5:**
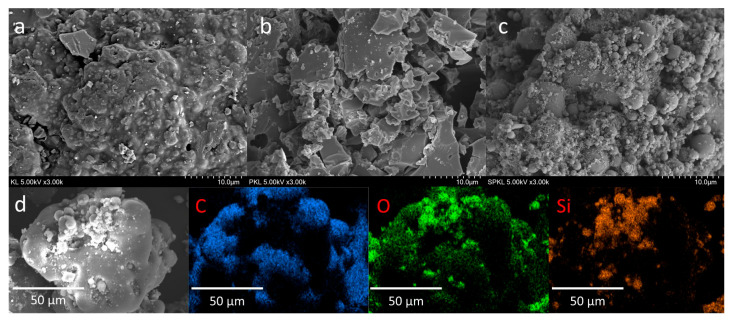
SEM images of (**a**) KL, (**b**) PKL, and (**c**) SPKL, and EDS mappings of the elements in the (**d**) SPKL.

SEM-EDS analysis was conducted to observe the morphological changes of KL, PKL, and SPKL, which were found to be clearly apparent. As shown in Figure 5, the particle surfaces of KL, which has not undergone any chemical treatment, showed no traces of special coating layers or attached nanoparticles. In contrast, the particle surfaces of PKL exhibited greater irregularity and a higher degree of deformation compared to those of KL, with some surfaces displaying signs of cracking and microporosity. This is attributed to the deformation of the lignin molecular structure and a decrease in intermolecular cohesion due to the binding of phenol molecules to KL. The surface of SPKL exhibits greater aggregation compared to PKL, characterized by the densely packed presence of spherical silica particles of varying sizes. Additionally, the EDS spectrum analysis of SPKL revealed C (61.40 wt%), O (30.55 wt%), and Si (7.30 wt%), clearly confirming that silica particles are chemically bonded to the lignin surface.

### 3.2. Evaluation of Flame Retardancy of PLA Composites

**Figure 6:**
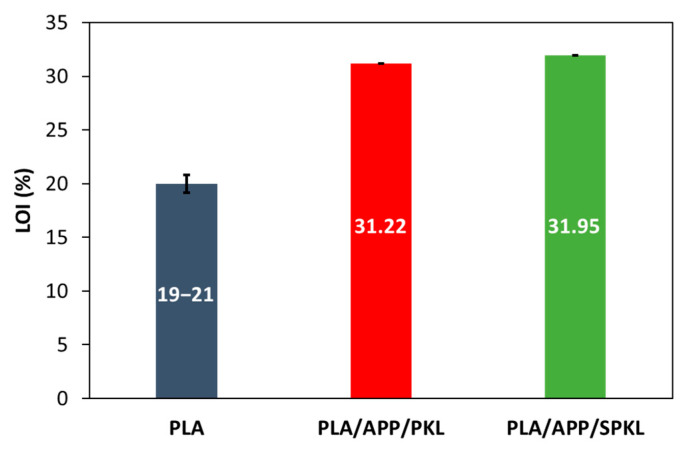
Results in LOI tests of PLA composites.

UL-94 vertical burning tests and limiting oxygen index (LOI) measurements were conducted to evaluate the flame retardancy of the composites, and the results were used to identify the optimal flame-retardant formulation for PLA (Figure 6 and Table 2).

Neat PLA ignited immediately in the UL-94 test, produced continuous molten drips, and ignited the cotton pad beneath, preventing any rating. The PLA/APP composite exhibited dripping and cotton ignition during the first 10 s of flame exposure but self-extinguished, confirming the baseline flame-retardant effect of APP. The PLA/APP/KL composite likewise showed cotton ignition but no dripping in the initial 10 s and self-extinguished, achieving a V-2 rating, indicating that lignin as a carbon source enhanced flame retardancy versus the APP-only system [12]. The PLA/APP/PKL composite exhibited no dripping during the first 10 s but produced 5–9 small droplets upon re-ignition, resulting in a V-1 rating. In contrast, the PLA/APP/SPKL composite showed no dripping at any stage and sustained a V-0 rating.

It is widely known that neat PLA has a limited oxygen index (LOI) of 19–21%; however, the PLA/APP/PKL and PLA/APP/SPKL composites prepared in this study achieved LOIs more than 30%. In particular, the PLA/APP/SPKL composite achieved an LOI of 31.95%, which is more than 50% higher than that of neat PLA, at 19–21%. The enhancement in LOI is ascribed to the supplementary hydroxyl groups, which encourage cross-linking with APP and augment condensed-phase flame-retardant performance through maximized char formation [34]. In particular, the highest LOI value was achieved by the PLA/APP/SPKL composite, which also maintained a V-0 rating. This is attributable to carbon contraction inhibition and enhanced barrier properties in the condensed phase of the silica hybrid [35].

### 3.3. Thermal Stability of Lignin and PLA Composites

Polymer composites incorporating lignin are known to enhance thermal properties such as thermal stability, and the use of two or more flame-retardant fillers often yields synergistic effects [36]. In this study, thermogravimetric analysis (TGA) was performed under a nitrogen and oxygen atmosphere to investigate the thermal degradation behavior of lignin and PLA composites due to lignin incorporation. The temperatures corresponding to 5% weight loss (T_5_), 50% weight loss (T_50_), and the maximum decomposition temperature (T_max_) were extracted to assess improvements in thermal stability.

**Figure 7:**
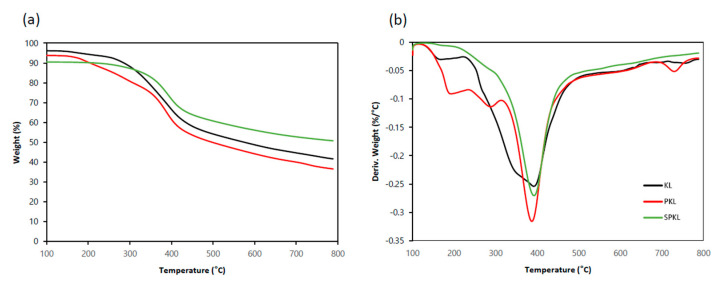
TG (**a**) and DTG (**b**) curves of flame retardants and KL, PKL, and SPKL under N_2_.

The thermal decomposition characteristics of three types of lignin (KL, PKL, and SPKL) were determined using TGA analysis to investigate the thermal decomposition behavior of lignin added as a flame retardant. As shown in Figure 7a, the thermal decomposition of KL occurs between approximately 200 °C and 600 °C, with the decomposition of aromatic rings and side chains constituting the predominant reactions during this temperature range. PKL, which underwent phenolation, exhibited thermal behavior like that previously reported in the studies [37,38]. In comparison with KL, PKL exhibited marginally diminished values for both T_5_ and T_50_. As shown in Table 3, KL had an onset temperature (T_onset_) of 286.05 °C, a T_max_ of 391.86 °C, and a final residue at 800 °C of 41.59 wt%. In contrast, the PKL T_onset_ was 299.40 °C, approximately 13 °C higher than KL, but T_max_ was 387.12 °C, 4.74 °C lower than KL’s, and the final residue was 36.54 wt%, a decrease of approximately 5 wt%. This is attributed to the partial decomposition of the lignin skeleton during the phenolation process, leading to low-molecular-weight formation, and the higher hydroxyl content, which facilitated the faster removal of volatile substances in the 200–300 °C temperature range. Conversely, SPKL, which was further silanized with PKL, exhibited significantly improved thermal stability. The T_onset_ of SPKL was 332.47 °C, an increase of approximately 46 °C compared to KL, indicating that the silica network effectively protected the low-molecular-weight lignin skeleton. Furthermore, the final residue of SPKL was found to be 50.75 wt%, representing an increase of over 9 wt% in comparison with KL. This finding suggests that not only did the organic lignin carbonize, but also the siloxane cross-linked structures that were formed during the silylation process remained as SiO_2_ during the process of pyrolysis. The results obtained from this study suggest that SPKL forms a mixed phase (char/inorganic network) by combining the inherent char of lignin with an inorganic silica network. This process maintains a higher residual rate during pyrolysis.

**Figure 8:**
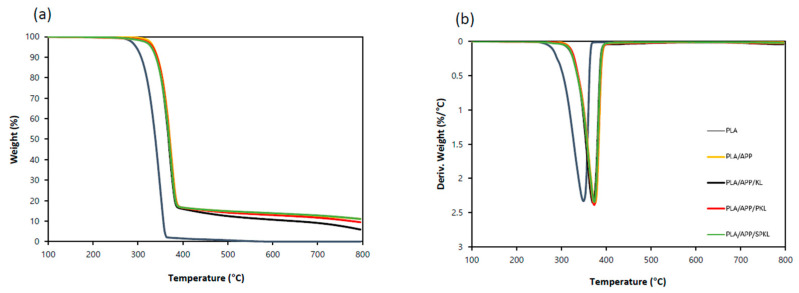
TG (**a**) and DTG (**b**) curves of flame retardants and PLA composites under N_2_.

As demonstrated in Figure 8, the TGA and DTG curves validate the thermal stability and thermal decomposition behavior in a nitrogen environment. It is apparent that the degradation profiles of the composites (PLA/APP, PLA/APP/KL, PLA/APP/PKL, and PLA/APP/SPKL) shift to higher temperatures in comparison to neat PLA, indicating enhanced thermal stability through chemical modification, including phenolation and silylation. As summarized in Table 4, neat PLA exhibits T_5_ = 295.95 °C, T_50_ = 338.80 °C, and T_max_ = 349.28 °C, with only 0.58% char remaining at 800 °C. Incorporation of APP raises T_5_ to 334.53 °C, confirming APP’s thermal stabilizing effect. The PLA/APP/KL composite, however, shows slightly lower T_5_ and T_max_ than PLA/APP, implying that unmodified lignin lowers the onset of degradation and promotes early char formation. Compared to PLA/APP/KL and PLA/APP/PKL, the PLA/APP/SPKL composite achieves the highest char yield (11.37 wt%) at 800 °C; the increased residue is attributed to the silylation treatment, which facilitates formation of a robust char layer that effectively suppresses release of combustible volatiles and inhibits flame propagation [18,39]. Moreover, the DTG peak corresponding to the maximum decomposition rate for PLA/APP/SPKL shifted from 349.28 °C (neat PLA) to 373.40 °C, further evidence that silylation enhances the thermal resistance of the composite.

**Figure 9:**
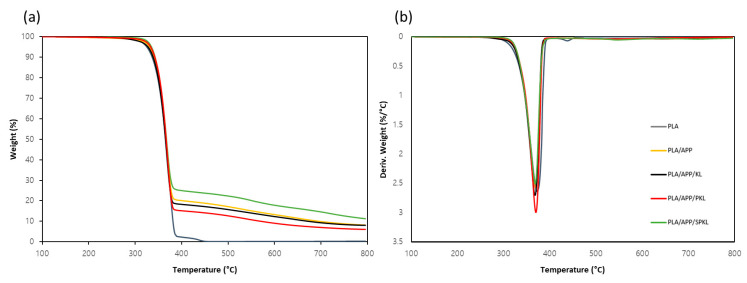
TG (**a**) and DTG (**b**) curves of flame retardants and PLA composites under air.

Figure 9 and Table 5 compare the thermal stability and pyrolysis behavior of the composites under air conditions that represent an actual fire. The neat PLA burned almost completely, leaving only 0.29 wt% residue. For PLA/APP, the T_5_ value decreased slightly to 331.24 °C versus nitrogen, and the final residue rose to 8.14 wt%, confirming that APP promotes char-layer formation in air, albeit with some loss of residue due to oxidative combustion. PLA/APP/KL yielded a T_5_ of 327.73 °C and 8.03 wt% residue, demonstrating that KL maintains thermal stability and char-forming capacity on par with APP alone. Phenolated lignin (PLA/APP/PKL) increased T_5_ to 333.34 °C, reflecting its higher –OH content, but the final residue fell to 6.04 wt% as volatile fragments underwent oxidative combustion. Most notably, PLA/APP/SPKL achieved the highest T_5_ (335.10 °C), the highest Tmax (367.99 °C), and the greatest residue (11.25 wt%) of all samples. This superior performance is attributed to the silica network’s ability to hinder lignin decomposition in an oxidizing atmosphere, thereby maximizing char-layer development. Overall, PLA/APP/SPKL exhibits the best flame-retardant performance under both nitrogen and air, underscoring the synergistic benefit of combining APP, phenolated lignin, and silica.

### 3.4. Analysis of Residual Char

**Figure 10:**
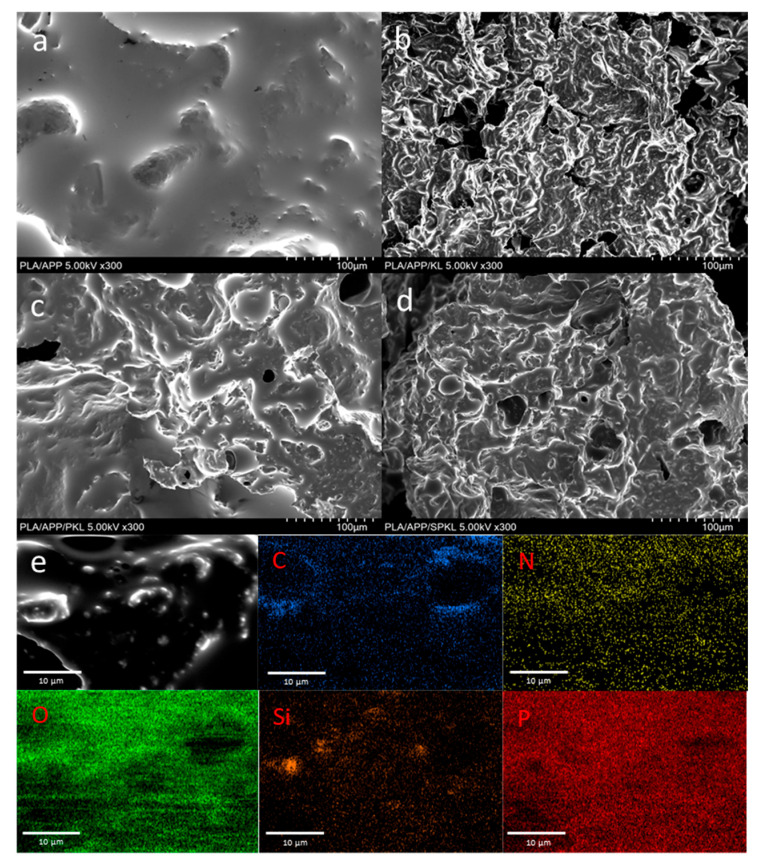
SEM images of (**a**) PLA/APP, (**b**) PLA/APP/KL, (**c**) PLA/APP/PKL, and (**d**) PLA/APP/SPKL, and EDS mappings of the elements in the (**e**) PLA/APP/SPKL.

Figure 10 presents SEM images of neat PLA and its composites after UL-94 testing (Figure 10a–d), along with an EDS elemental map of the PLA/APP/SPKL surface (Figure 10e).

The neat PLA produced no char and showed almost no residual material, while PLA/APP (Figure 10a) exhibited a smooth, pore-free fracture surface—consistent with its minimal char formation in the UL-94 test. Upon lignin incorporation, all three composites developed distinct char morphologies. PLA/APP/KL (Figure 10b) displayed a rough surface with thick, agglomerated char clusters and locally dense deposits. In contrast, PLA/APP/PKL (Figure 10c) formed a thinner, more uniform char layer with numerous pores and reduced aggregation compared to KL. PLA/APP/SPKL (Figure 10d) combined a smooth macroscopic appearance with a densely microporous char network, indicating that the TEOS-derived silica had reinforced the char layer to create a continuous protective barrier.

The EDS maps of PLA/APP/SPKL (Figure 10e) confirm that C and O are homogeneously distributed throughout the char, that N and P originating from the ammonium polyphosphate flame retardant uniformly cover the composite surface, and that Si is evenly dispersed, verifying the formation of a silica network. This synergistic integration of lignin, APP, and silica yields a cohesive, mechanically robust char layer that underpins the enhanced flame-retardant performance observed in both LOI and UL-94 measurements.

### 3.5. Mechanical Properties of PLA Composites

**Figure 11:**
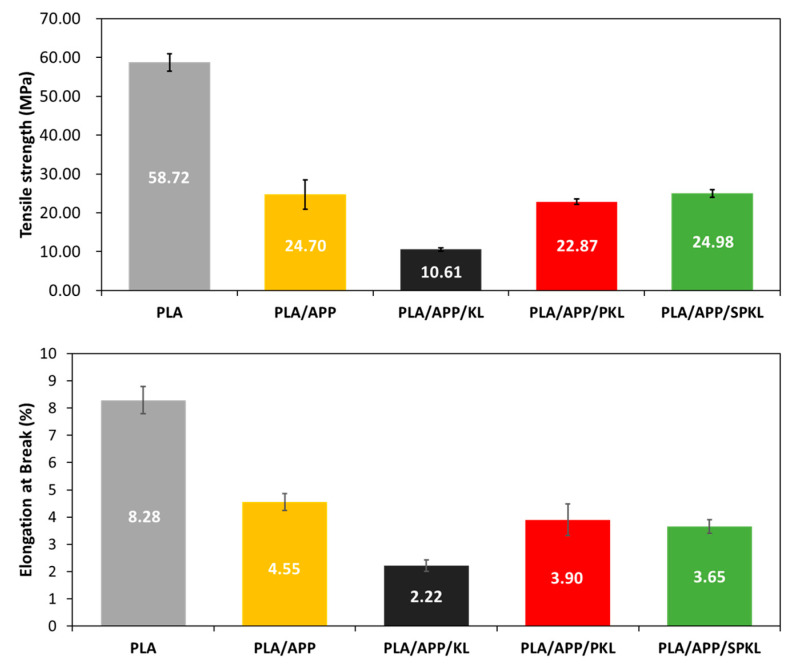
Tensile strength and elongation of break of PLA composites with different compositions.

Degradation of mechanical properties induced by flame retardant addition is a well-known challenge, and in this study we evaluated the changes in PLA composite performance resulting from the incorporation of APP and lignin-based additives [40]. The tensile strength and elongation at break were chosen to evaluate the composites’ mechanical properties, and the results for each formulation are shown in Figure 11. It is generally reported that adding APP and significant amounts of lignin to matrices such as PLA causes a substantial drop in tensile strength and elongation at break when imparting flame retardancy, because the dispersed APP and lignin particles weaken interfacial interactions within the PLA matrix and disrupt its continuous phase formation [41,42]. Silica-based flame retardants, however, are known to help recover mechanical strength, and since phenolation was performed to achieve higher degrees of silylation, we investigated the effects of both silylation and phenolation on tensile strength.

As demonstrated in Figure 11, the composite materials comprising PLA/APP, PLA/APP/KL, PLA/APP/PKL, and PLA/APP/SPKL exhibited a substantial decrease in tensile strength in comparison to neat PLA. In particular, the PLA/APP/KL composite, which was composed with lignin to enhance flame retardancy, exhibited an additional decrease in tensile strength. However, when compared to the PLA/APP/KL system with unmodified lignin, the PLA/APP/PKL composite with chemically modified lignin, exhibiting superior flame retardancy, demonstrated a 115.6% higher tensile strength and a 75.7% increase in elongation at break. The findings indicate that while the decrease in tensile strength resulting from the addition of APP is inevitable, the reduction in mechanical properties caused by lignin addition can be mitigated through lignin modification. It has been suggested that this mitigation can be attributed to phenolation’s reduction of lignin’s molecular-weight distribution and the formation of hydrogen bonds between the newly abundant hydroxyl groups on the lignin chains and the carbonyl groups of PLA. This enhanced interfacial activity and compatibility is a significant finding in this study [43]. Furthermore, although the elongation at break of the PLA/APP/SPKL composite decreased slightly, it remained at a similar level to that of PLA/APP/PKL, while its tensile strength increased by 9.22% compared to PLA/APP/PKL. This is attributed to the uniform dispersion of silica particles bound to the phenolated lignin within the PLA matrix, thereby reinforcing the composite’s tensile properties [44]. The findings demonstrate that sequential addition of phenol and silane groups to lignin can enhance the mechanical performance of flame-retardant PLA composites [45].

## 4. Conclusions

The objective of this study was to enhance the flame retardancy of PLA, a commercial biodegradable plastic, through the utilization of a phosphate-based additive, namely APP, and lignin, which is a predominant component of lignocellulosic biomass. To enhance the flame retardancy of PLA composites while concomitantly minimizing the decrease in mechanical properties, phenolation and silylation were performed on lignin. Subsequently, chemically modified lignin and APP were applied to PLA to fabricate biodegradable, flame-retardant composites. These composites were then subjected to a range of evaluations.

The results of the flame retardancy evaluation of biodegradable flame-retardant plastics using a modified lignin and APP-based intumescent flame-retardant system showed that no flame retardancy enhancement effect was observed with the addition of chemically unmodified KL, and only a limited flame retardancy enhancement effect was observed with PKL. These results suggest that lignin acts solely as a carbon source and does not impart excellent flame retardancy to the composite material. However, upon the application of SPKL, a synergistic effect emerged between APP and silica, yielding optimal flame retardancy. TGA analysis confirmed that the addition of APP enhanced the thermal stability of the composite. In the case of the composite with lignin added, it was confirmed that lignin contributed to the enhancement of thermal stability by lowering the initial decomposition temperature and promoting the formation of a carbonized layer. It is notable that phenolated and silylated lignin (SPKL) itself retained 50.75 wt% char at 800 °C, whereas when incorporated into the composite, it yielded 11.37 wt% under a nitrogen atmosphere and 11.25 wt% in air, highlighting its rapid carbonization layer-forming capability. It is hypothesized that the high carbon residue ratio will contribute to the effective suppression of flammable gas emission.

As is well known, when APP and lignin were used simultaneously to impart flame retardancy to PLA, the tensile strength of the PLA composite decreased significantly; however, this decrease was mitigated by the phenolation of lignin. This phenomenon is attributed to the enhanced interface interaction and compatibility resulting from the formation of hydrogen bonds between PLA and phenolated lignin. Partial improvement in tensile strength was observed with the addition of silica, which is estimated to be due to the synergistic effect supported by the uniform dispersion of silica particles bound to silylated lignin within the PLA matrix.

In this study, the flame retardancy and mechanical properties of PLA composites were enhanced through the phenolation and silylation of lignin. Furthermore, a biodegradable flame-retardant composite with high thermal stability and good mechanical properties was fabricated by applying chemically modified lignin and APP, a phosphate-based additive, to PLA. However, it should be noted that, although PLA is inherently compostable, the incorporation of non-biodegradable additives such as APP and silylated lignin significantly alters its degradation behavior. Consequently, it can be deduced that the resulting composite materials should not be assumed to be biodegradable under standard composting conditions. It may be necessary for these materials to be exposed to specialized degradation environments, such as high-silicate or mineral-rich soils.

## Figures and Tables

**Table 1:** Formulation of PLA composites (wt%).

Sample	PLA	APP	KL	PKL	SPKL
PLA	100	0	0	0	0
PLA/APP	80	20	0	0	0
PLA/APP/KL	80	14	6	0	0
PLA/APP/PKL	80	14	0	6	0
PLA/APP/SPKL	80	14	0	0	6

**Table 2:** Results in UL-94 vertical burning tests of PLA composites.

Sample	PLA	PLA/APP	PLA/APP/KL	PLA/APP/PKL	PLA/APP/SPKL
Rating	NR	V-2	V-2	V-1	V-0
Dripping *	Yes/Yes	Yes/Yes	No/Yes	No/Yes	No/No
Ignition of the cotton	Yes	Yes	Yes	No	No

* First ignition/second ignition: Presence or absence of molten material falling for 10 s.

**Table 3:** T_onset_, T_max_, and residue at 800 °C (wt%) data of KL, PKL, and SPKL.

Sample	T_onset_ (°C)	T_max_ (°C)	Residue at 800 °C (wt%)
KL	286.05	391.86	41.59
PKL	299.40	387.12	36.54
SPKL	332.47	392.40	50.75

**Table 4:** T_5_, T_50_, T_max_, and residue at 800 °C (wt%) data of PLA composites with various compositions in a nitrogen environment.

Sample	T_5_ (°C)	T_50_ (°C)	T_max_ (°C)	Residue at 800 °C (wt%)
PLA	295.65	338.70	349.28	0.58
PLA/APP	334.53	371.00	375.25	11.40
PLA/APP/KL	330.12	367.98	370.64	6.21
PLA/APP/PKL	332.70	370.04	373.40	9.85
PLA/APP/SPKL	329.46	369.26	373.40	11.37

**Table 5:** T_5_, T_50_, T_max_, and residue at 800 °C (wt%) data of PLA composites with various compositions in an air environment.

Sample	T_5_ (°C)	T_50_ (°C)	T_max_ (°C)	Residue at 800 °C (wt%)
PLA	325.15	365.57	372.22	0.29
PLA/APP	331.24	366.16	368.63	8.14
PLA/APP/KL	327.73	365.09	367.97	8.03
PLA/APP/PKL	333.34	366.57	369.84	6.04
PLA/APP/SPKL	335.10	367.32	367.99	11.25

## Data Availability

Data are contained within the article.
